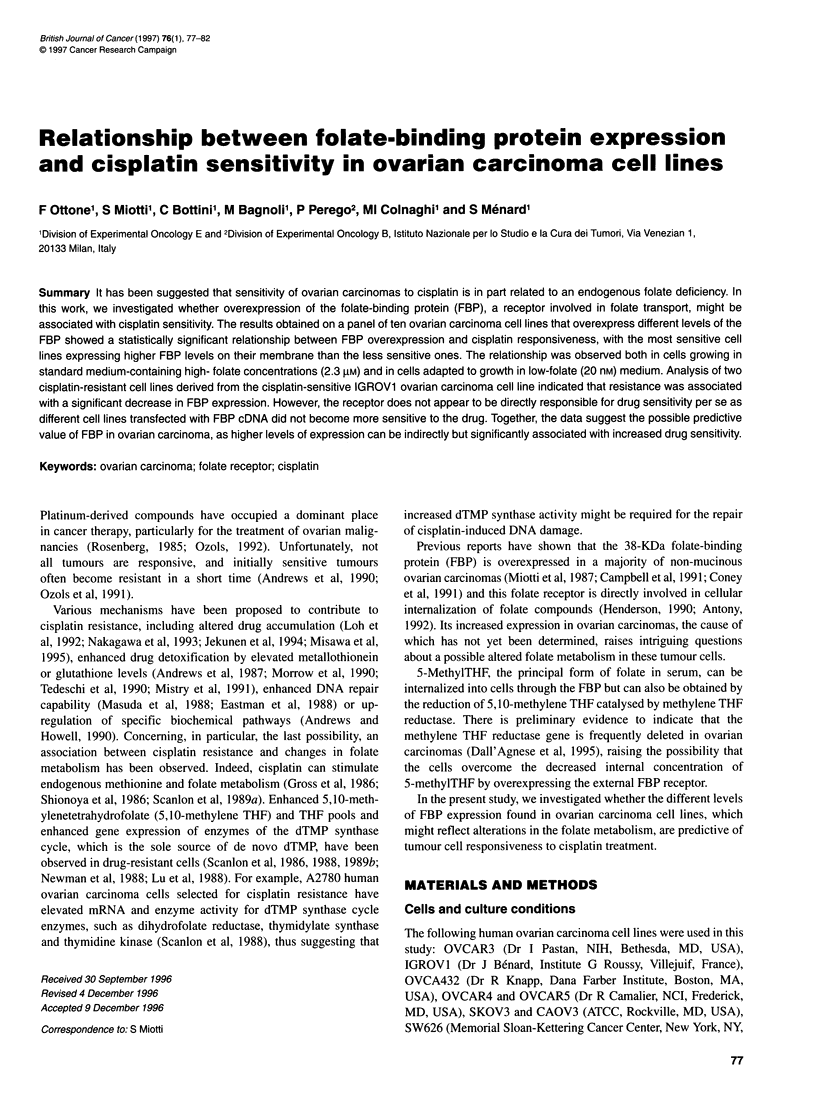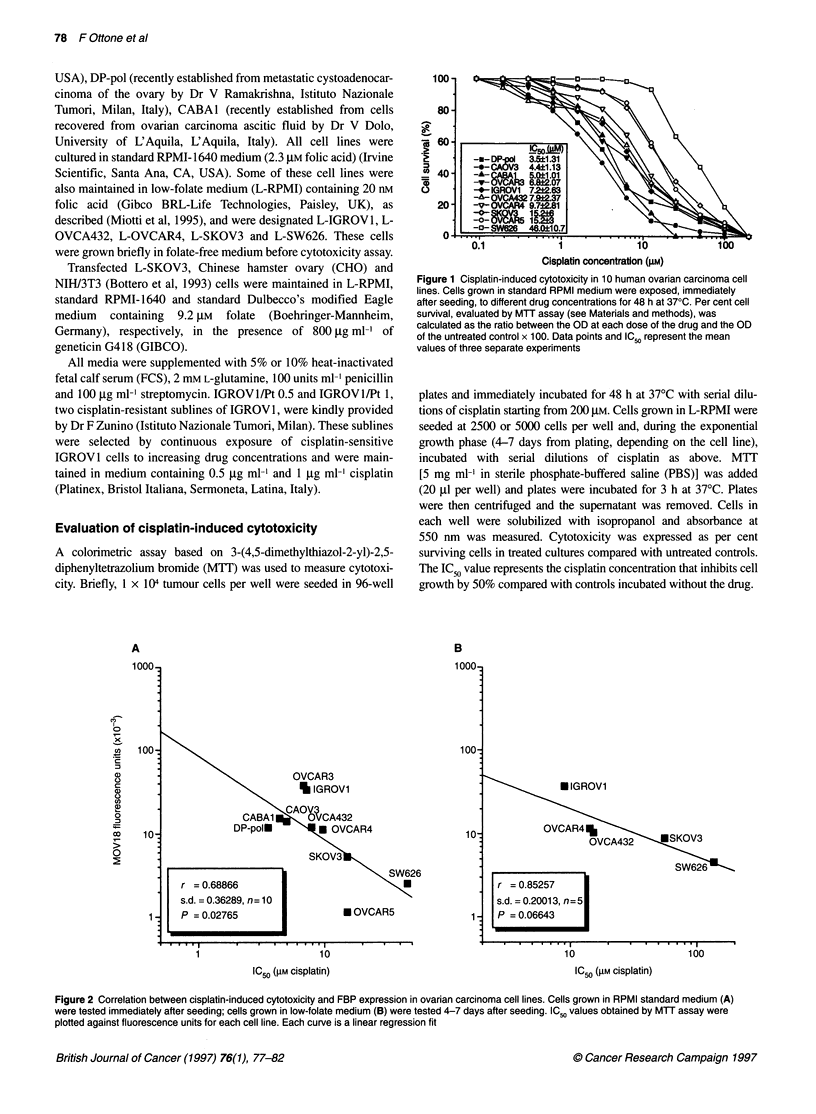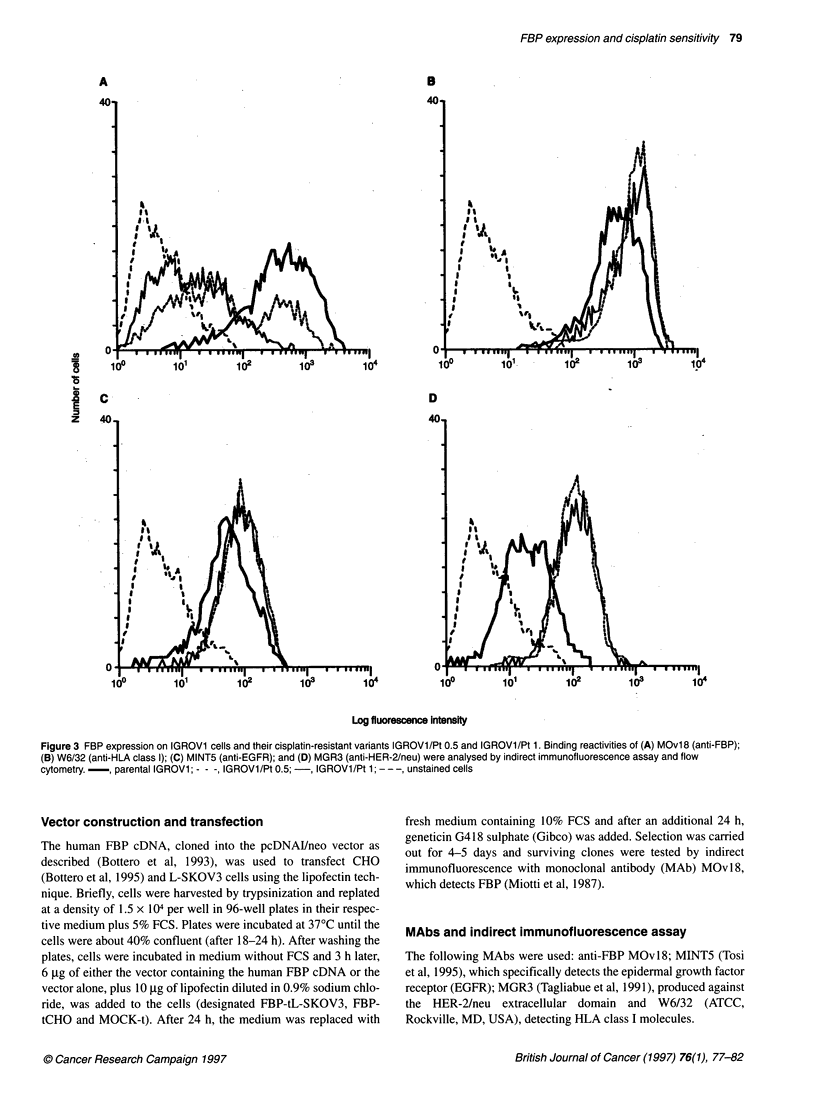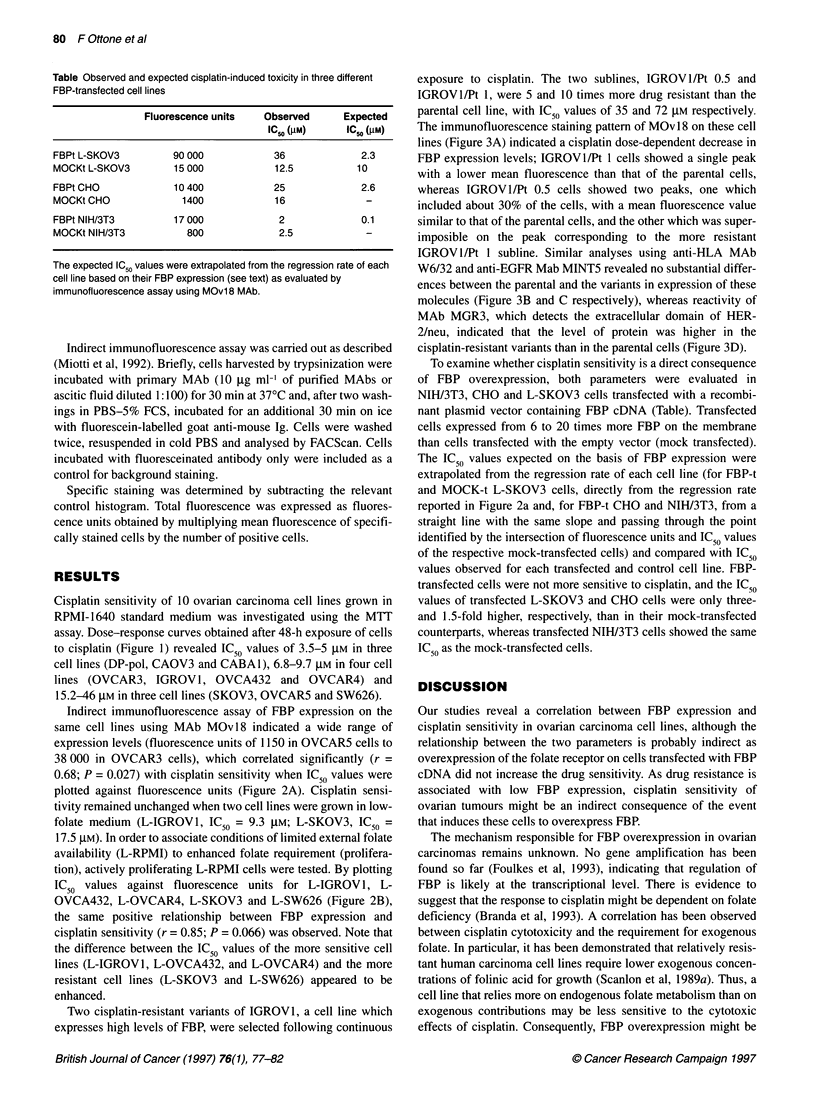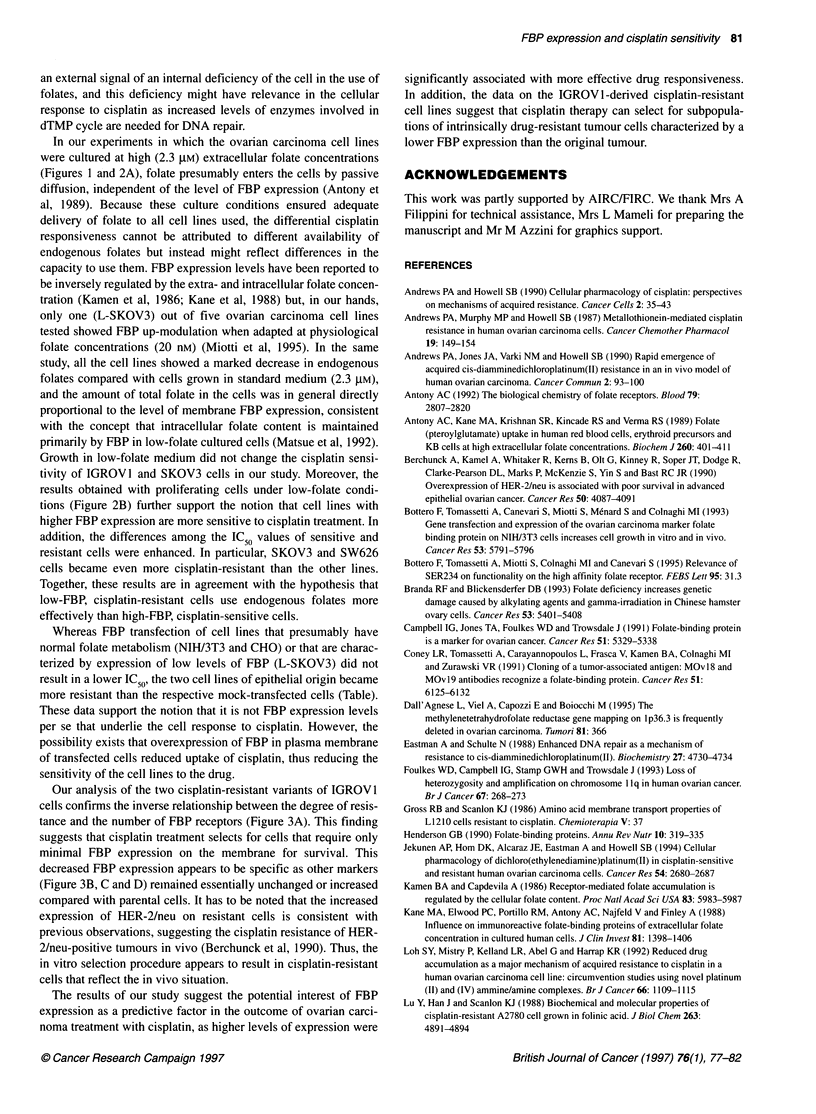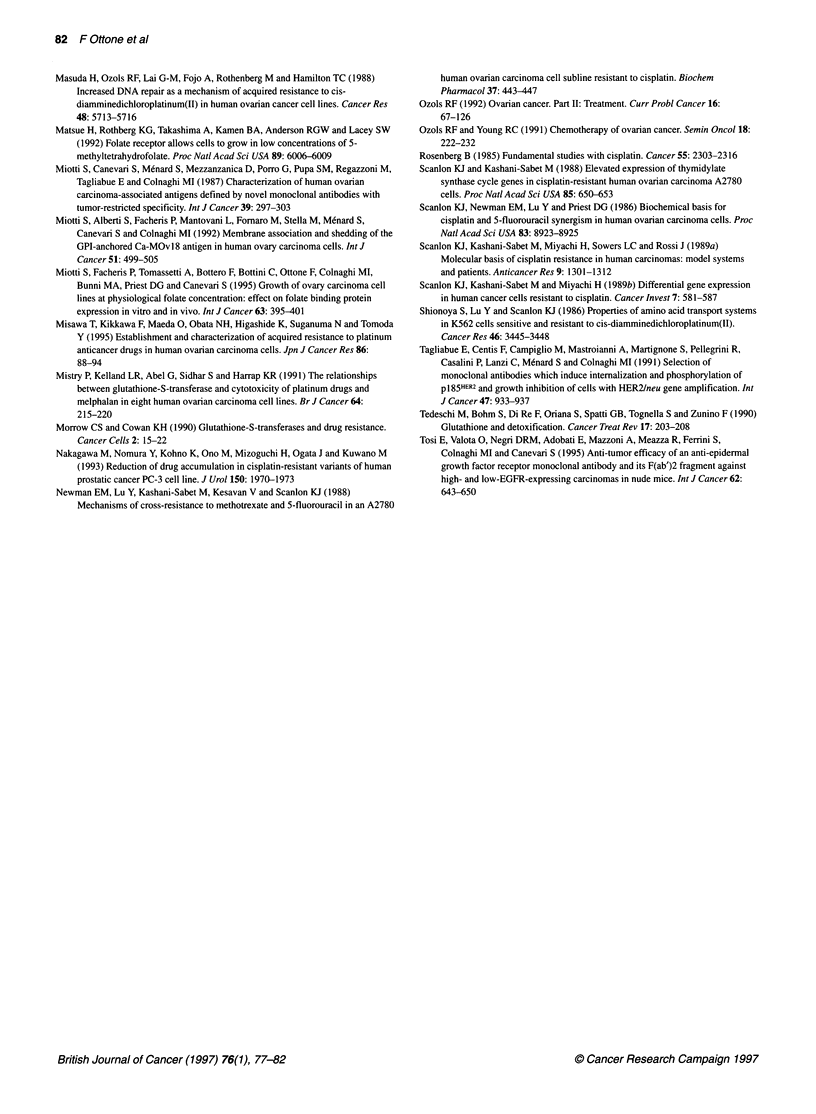# Relationship between folate-binding protein expression and cisplatin sensitivity in ovarian carcinoma cell lines.

**DOI:** 10.1038/bjc.1997.339

**Published:** 1997

**Authors:** F. Ottone, S. Miotti, C. Bottini, M. Bagnoli, P. Perego, M. I. Colnaghi, S. MÃ©nard

**Affiliations:** Division of Experimental Oncology E, Istituto Nazionale per lo Studio e la Cura dei Tumori, Milan, Italy.

## Abstract

It has been suggested that sensitivity of ovarian carcinomas to cisplatin is in part related to an endogenous folate deficiency. In this work, we investigated whether overexpression of the folate-binding protein (FBP), a receptor involved in folate transport, might be associated with cisplatin sensitivity. The results obtained on a panel of ten ovarian carcinoma cell lines that overexpress different levels of the FBP showed a statistically significant relationship between FBP overexpression and cisplatin responsiveness, with the most sensitive cell lines expressing higher FBP levels on their membrane than the less sensitive ones. The relationship was observed both in cells growing in standard medium-containing high-folate concentrations (2.3 microM) and in cells adapted to growth in low-folate (20 nM) medium. Analysis of two cisplatin-resistant cell lines derived from the cisplatin-sensitive IGROV1 ovarian carcinoma cell line indicated that resistance was associated with a significant decrease in FBP expression. However, the receptor does not appear to be directly responsible for drug sensitivity per se as different cell lines transfected with FBP cDNA did not become more sensitive to the drug. Together, the data suggest the possible predictive value of FBP in ovarian carcinoma, as higher levels of expression can be indirectly but significantly associated with increased drug sensitivity.


British Journal of Cancer (1997) 76(1), 77–82
© 1997 Cancer Research Campaign

### Relationship between folate-binding protein expression and cisplatin sensitivity in ovarian carcinoma cell lines

**F Ottone[1], S Miotti[1], C Bottini[1], M Bagnoli[1], P Perego[2], MI Colnaghi[1] and S Ménard[1]**

[1]Division of Experimental Oncology E and [2]Division of Experimental Oncology B, Istituto Nazionale per lo Studio e la Cura dei Tumori, Via Venezian 1, 20133 Milan, Italy

**Summary** It has been suggested that sensitivity of ovarian carcinomas to cisplatin is in part related to an endogenous folate deficiency. In this work, we investigated whether overexpression of the folate-binding protein (FBP), a receptor involved in folate transport, might be associated with cisplatin sensitivity. The results obtained on a panel of ten ovarian carcinoma cell lines that overexpress different levels of the FBP showed a statistically significant relationship between FBP overexpression and cisplatin responsiveness, with the most sensitive cell lines expressing higher FBP levels on their membrane than the less sensitive ones. The relationship was observed both in cells growing in standard medium-containing high- folate concentrations (2.3 $\mu$M) and in cells adapted to growth in low-folate (20 nM) medium. Analysis of two cisplatin-resistant cell lines derived from the cisplatin-sensitive IGROV1 ovarian carcinoma cell line indicated that resistance was associated with a significant decrease in FBP expression. However, the receptor does not appear to be directly responsible for drug sensitivity per se as different cell lines transfected with FBP cDNA did not become more sensitive to the drug. Together, the data suggest the possible predictive value of FBP in ovarian carcinoma, as higher levels of expression can be indirectly but significantly associated with increased drug sensitivity.

**Keywords:** ovarian carcinoma; folate receptor; cisplatin

Platinum-derived compounds have occupied a dominant place in cancer therapy, particularly for the treatment of ovarian malignancies (Rosenberg, 1985; Ozols, 1992). Unfortunately, not all tumours are responsive, and initially sensitive tumours often become resistant in a short time (Andrews et al, 1990; Ozols et al, 1991).

Various mechanisms have been proposed to contribute to cisplatin resistance, including altered drug accumulation (Loh et al, 1992; Nakagawa et al, 1993; Jekunen et al, 1994; Misawa et al, 1995), enhanced drug detoxification by elevated metallothionein or glutathione levels (Andrews et al, 1987; Morrow et al, 1990; Tedeschi et al, 1990; Mistry et al, 1991), enhanced DNA repair capability (Masuda et al, 1988; Eastman et al, 1988) or up-regulation of specific biochemical pathways (Andrews and Howell, 1990). Concerning, in particular, the last possibility, an association between cisplatin resistance and changes in folate metabolism has been observed. Indeed, cisplatin can stimulate endogenous methionine and folate metabolism (Gross et al, 1986; Shionoya et al, 1986; Scanlon et al, 1989*a*). Enhanced 5,10-methylenetetrahydrofolate (5,10-methylene THF) and THF pools and enhanced gene expression of enzymes of the dTMP synthase cycle, which is the sole source of de novo dTMP, have been observed in drug-resistant cells (Scanlon et al, 1986, 1988, 1989*b*; Newman et al, 1988; Lu et al, 1988). For example, A2780 human ovarian carcinoma cells selected for cisplatin resistance have elevated mRNA and enzyme activity for dTMP synthase cycle enzymes, such as dihydrofolate reductase, thymidylate synthase and thymidine kinase (Scanlon et al, 1988), thus suggesting that increased dTMP synthase activity might be required for the repair of cisplatin-induced DNA damage.

*Received 30 September 1996*
*Revised 4 December 1996*
*Accepted 9 December 1996*

*Correspondence to:* S Miotti

Previous reports have shown that the 38-KDa folate-binding protein (FBP) is overexpressed in a majority of non-mucinous ovarian carcinomas (Miotti et al, 1987; Campbell et al, 1991; Coney et al, 1991) and this folate receptor is directly involved in cellular internalization of folate compounds (Henderson, 1990; Antony, 1992). Its increased expression in ovarian carcinomas, the cause of which has not yet been determined, raises intriguing questions about a possible altered folate metabolism in these tumour cells.

5-MethylTHF, the principal form of folate in serum, can be internalized into cells through the FBP but can also be obtained by the reduction of 5,10-methylene THF catalysed by methylene THF reductase. There is preliminary evidence to indicate that the methylene THF reductase gene is frequently deleted in ovarian carcinomas (Dall'Agnese et al, 1995), raising the possibility that the cells overcome the decreased internal concentration of 5-methylTHF by overexpressing the external FBP receptor.

In the present study, we investigated whether the different levels of FBP expression found in ovarian carcinoma cell lines, which might reflect alterations in the folate metabolism, are predictive of tumour cell responsiveness to cisplatin treatment.

### MATERIALS AND METHODS

### Cells and culture conditions

The following human ovarian carcinoma cell lines were used in this study: OVCAR3 (Dr I Pastan, NIH, Bethesda, MD, USA), IGROV1 (Dr J Bénard, Institute G Roussy, Villejuif, France), OVCA432 (Dr R Knapp, Dana Farber Institute, Boston, MA, USA), OVCAR4 and OVCAR5 (Dr R Camalier, NCI, Frederick, MD, USA), SKOV3 and CAOV3 (ATCC, Rockville, MD, USA), SW626 (Memorial Sloan-Kettering Cancer Center, New York, NY,

USA), DP-pol (recently established from metastatic cystoadenocarcinoma of the ovary by Dr V Ramakrishna, Istituto Nazionale Tumori, Milan, Italy), CABA1 (recently established from cells recovered from ovarian carcinoma ascitic fluid by Dr V Dolo, University of L'Aquila, L'Aquila, Italy). All cell lines were cultured in standard RPMI-1640 medium (2.3 μM folic acid) (Irvine Scientific, Santa Ana, CA, USA). Some of these cell lines were also maintained in low-folate medium (L-RPMI) containing 20 nM folic acid (Gibco BRL-Life Technologies, Paisley, UK), as described (Miotti et al, 1995), and were designated L-IGROV1, L-OVCA432, L-OVCAR4, L-SKOV3 and L-SW626. These cells were grown briefly in folate-free medium before cytotoxicity assay.

Transfected L-SKOV3, Chinese hamster ovary (CHO) and NIH/3T3 (Bottero et al, 1993) cells were maintained in L-RPMI, standard RPMI-1640 and standard Dulbecco's modified Eagle medium containing 9.2 μM folate (Boehringer-Mannheim, Germany), respectively, in the presence of 800 μg $ml^{-1}$ of geneticin G418 (GIBCO).

All media were supplemented with 5% or 10% heat-inactivated fetal calf serum (FCS), 2 mM L-glutamine, 100 units $ml^{-1}$ penicillin and 100 μg $ml^{-1}$ streptomycin. IGROV1/Pt 0.5 and IGROV1/Pt 1, two cisplatin-resistant sublines of IGROV1, were kindly provided by Dr F Zunino (Istituto Nazionale Tumori, Milan). These sublines were selected by continuous exposure of cisplatin-sensitive IGROV1 cells to increasing drug concentrations and were maintained in medium containing 0.5 μg $ml^{-1}$ and 1 μg $ml^{-1}$ cisplatin (Platinex, Bristol Italiana, Sermoneta, Latina, Italy).

### Evaluation of cisplatin-induced cytotoxicity

A colorimetric assay based on 3-(4,5-dimethylthiazol-2-yl)-2,5-diphenyltetrazolium bromide (MTT) was used to measure cytotoxicity. Briefly, 1 × $10^4$ tumour cells per well were seeded in 96-well plates and immediately incubated for 48 h at 37°C with serial dilutions of cisplatin starting from 200 μM. Cells grown in L-RPMI were seeded at 2500 or 5000 cells per well and, during the exponential growth phase (4–7 days from plating, depending on the cell line), incubated with serial dilutions of cisplatin as above. MTT [5 mg $ml^{-1}$ in sterile phosphate-buffered saline (PBS)] was added (20 μl per well) and plates were incubated for 3 h at 37°C. Plates were then centrifuged and the supernatant was removed. Cells in each well were solubilized with isopropanol and absorbance at 550 nm was measured. Cytotoxicity was expressed as per cent surviving cells in treated cultures compared with untreated controls. The $IC_{50}$ value represents the cisplatin concentration that inhibits cell growth by 50% compared with controls incubated without the drug.

**Figure 1** Cisplatin-induced cytotoxicity in 10 human ovarian carcinoma cell lines. Cells grown in standard RPMI medium were exposed, immediately after seeding, to different drug concentrations for 48 h at 37°C. Per cent cell survival, evaluated by MTT assay (see Materials and methods), was calculated as the ratio between the OD at each dose of the drug and the OD of the untreated control × 100. Data points and $IC_{50}$ represent the mean values of three separate experiments

**Figure 2** Correlation between cisplatin-induced cytotoxicity and FBP expression in ovarian carcinoma cell lines. Cells grown in RPMI standard medium (**A**) were tested immediately after seeding; cells grown in low-folate medium (**B**) were tested 4–7 days after seeding. $IC_{50}$ values obtained by MTT assay were plotted against fluorescence units for each cell line. Each curve is a linear regression fit

© Cancer Research Campaign 1997

**Figure 3** FBP expression on IGROV1 cells and their cisplatin-resistant variants IGROV1/Pt 0.5 and IGROV1/Pt 1. Binding reactivities of (**A**) MOv18 (anti-FBP); (**B**) W6/32 (anti-HLA class I); (**C**) MINT5 (anti-EGFR); and (**D**) MGR3 (anti-HER-2/neu) were analysed by indirect immunofluorescence assay and flow cytometry. ▬▬, parental IGROV1; - - -, IGROV1/Pt 0.5; —, IGROV1/Pt 1; – – –, unstained cells

### Vector construction and transfection

The human FBP cDNA, cloned into the pcDNAI/neo vector as described (Bottero et al, 1993), was used to transfect CHO (Bottero et al, 1995) and L-SKOV3 cells using the lipofectin technique. Briefly, cells were harvested by trypsinization and replated at a density of $1.5 \times 10^4$ per well in 96-well plates in their respective medium plus 5% FCS. Plates were incubated at 37°C until the cells were about 40% confluent (after 18–24 h). After washing the plates, cells were incubated in medium without FCS and 3 h later, 6 µg of either the vector containing the human FBP cDNA or the vector alone, plus 10 µg of lipofectin diluted in 0.9% sodium chloride, was added to the cells (designated FBP-tL-SKOV3, FBP-tCHO and MOCK-t). After 24 h, the medium was replaced with fresh medium containing 10% FCS and after an additional 24 h, geneticin G418 sulphate (Gibco) was added. Selection was carried out for 4–5 days and surviving clones were tested by indirect immunofluorescence with monoclonal antibody (MAb) MOv18, which detects FBP (Miotti et al, 1987).

### MAbs and indirect immunofluorescence assay

The following MAbs were used: anti-FBP MOv18; MINT5 (Tosi et al, 1995), which specifically detects the epidermal growth factor receptor (EGFR); MGR3 (Tagliabue et al, 1991), produced against the HER-2/neu extracellular domain and W6/32 (ATCC, Rockville, MD, USA), detecting HLA class I molecules.

© Cancer Research Campaign 1997

**Table** Observed and expected cisplatin-induced toxicity in three different FBP-transfected cell lines

| | Fluorescence units | Observed $IC_{50}$ (μM) | Expected $IC_{50}$ (μM) |
|---|---|---|---|
| FBPt L-SKOV3 | 90 000 | 36 | 2.3 |
| MOCKt L-SKOV3 | 15 000 | 12.5 | 10 |
| FBPt CHO | 10 400 | 25 | 2.6 |
| MOCKt CHO | 1400 | 16 | – |
| FBPt NIH/3T3 | 17 000 | 2 | 0.1 |
| MOCKt NIH/3T3 | 800 | 2.5 | – |

The expected $IC_{50}$ values were extrapolated from the regression rate of each cell line based on their FBP expression (see text) as evaluated by immunofluorescence assay using MOv18 MAb.

Indirect immunofluorescence assay was carried out as described (Miotti et al, 1992). Briefly, cells harvested by trypsinization were incubated with primary MAb (10 μg $ml^{-1}$ of purified MAbs or ascitic fluid diluted 1:100) for 30 min at 37°C and, after two washings in PBS–5% FCS, incubated for an additional 30 min on ice with fluorescein-labelled goat anti-mouse Ig. Cells were washed twice, resuspended in cold PBS and analysed by FACScan. Cells incubated with fluoresceinated antibody only were included as a control for background staining.

Specific staining was determined by subtracting the relevant control histogram. Total fluorescence was expressed as fluorescence units obtained by multiplying mean fluorescence of specifically stained cells by the number of positive cells.

### RESULTS

Cisplatin sensitivity of 10 ovarian carcinoma cell lines grown in RPMI-1640 standard medium was investigated using the MTT assay. Dose–response curves obtained after 48-h exposure of cells to cisplatin (Figure 1) revealed $IC_{50}$ values of 3.5–5 μM in three cell lines (DP-pol, CAOV3 and CABA1), 6.8–9.7 μM in four cell lines (OVCAR3, IGROV1, OVCA432 and OVCAR4) and 15.2–46 μM in three cell lines (SKOV3, OVCAR5 and SW626).

Indirect immunofluorescence assay of FBP expression on the same cell lines using MAb MOv18 indicated a wide range of expression levels (fluorescence units of 1150 in OVCAR5 cells to 38 000 in OVCAR3 cells), which correlated significantly ($r$ = 0.68; $P$ = 0.027) with cisplatin sensitivity when $IC_{50}$ values were plotted against fluorescence units (Figure 2A). Cisplatin sensitivity remained unchanged when two cell lines were grown in low-folate medium (L-IGROV1, $IC_{50}$ = 9.3 μM; L-SKOV3, $IC_{50}$ = 17.5 μM). In order to associate conditions of limited external folate availability (L-RPMI) to enhanced folate requirement (proliferation), actively proliferating L-RPMI cells were tested. By plotting $IC_{50}$ values against fluorescence units for L-IGROV1, L-OVCA432, L-OVCAR4, L-SKOV3 and L-SW626 (Figure 2B), the same positive relationship between FBP expression and cisplatin sensitivity ($r$ = 0.85; $P$ = 0.066) was observed. Note that the difference between the $IC_{50}$ values of the more sensitive cell lines (L-IGROV1, L-OVCA432, and L-OVCAR4) and the more resistant cell lines (L-SKOV3 and L-SW626) appeared to be enhanced.

Two cisplatin-resistant variants of IGROV1, a cell line which expresses high levels of FBP, were selected following continuous exposure to cisplatin. The two sublines, IGROV1/Pt 0.5 and IGROV1/Pt 1, were 5 and 10 times more drug resistant than the parental cell line, with $IC_{50}$ values of 35 and 72 μM respectively. The immunofluorescence staining pattern of MOv18 on these cell lines (Figure 3A) indicated a cisplatin dose-dependent decrease in FBP expression levels; IGROV1/Pt 1 cells showed a single peak with a lower mean fluorescence than that of the parental cells, whereas IGROV1/Pt 0.5 cells showed two peaks, one which included about 30% of the cells, with a mean fluorescence value similar to that of the parental cells, and the other which was superimposible on the peak corresponding to the more resistant IGROV1/Pt 1 subline. Similar analyses using anti-HLA MAb W6/32 and anti-EGFR Mab MINT5 revealed no substantial differences between the parental and the variants in expression of these molecules (Figure 3B and C respectively), whereas reactivity of MAb MGR3, which detects the extracellular domain of HER-2/neu, indicated that the level of protein was higher in the cisplatin-resistant variants than in the parental cells (Figure 3D).

To examine whether cisplatin sensitivity is a direct consequence of FBP overexpression, both parameters were evaluated in NIH/3T3, CHO and L-SKOV3 cells transfected with a recombinant plasmid vector containing FBP cDNA (Table). Transfected cells expressed from 6 to 20 times more FBP on the membrane than cells transfected with the empty vector (mock transfected). The $IC_{50}$ values expected on the basis of FBP expression were extrapolated from the regression rate of each cell line (for FBP-t and MOCK-t L-SKOV3 cells, directly from the regression rate reported in Figure 2a and, for FBP-t CHO and NIH/3T3, from a straight line with the same slope and passing through the point identified by the intersection of fluorescence units and $IC_{50}$ values of the respective mock-transfected cells) and compared with $IC_{50}$ values observed for each transfected and control cell line. FBP-transfected cells were not more sensitive to cisplatin, and the $IC_{50}$ values of transfected L-SKOV3 and CHO cells were only three- and 1.5-fold higher, respectively, than in their mock-transfected counterparts, whereas transfected NIH/3T3 cells showed the same $IC_{50}$ as the mock-transfected cells.

### DISCUSSION

Our studies reveal a correlation between FBP expression and cisplatin sensitivity in ovarian carcinoma cell lines, although the relationship between the two parameters is probably indirect as overexpression of the folate receptor on cells transfected with FBP cDNA did not increase the drug sensitivity. As drug resistance is associated with low FBP expression, cisplatin sensitivity of ovarian tumours might be an indirect consequence of the event that induces these cells to overexpress FBP.

The mechanism responsible for FBP overexpression in ovarian carcinomas remains unknown. No gene amplification has been found so far (Foulkes et al, 1993), indicating that regulation of FBP is likely at the transcriptional level. There is evidence to suggest that the response to cisplatin might be dependent on folate deficiency (Branda et al, 1993). A correlation has been observed between cisplatin cytotoxicity and the requirement for exogenous folate. In particular, it has been demonstrated that relatively resistant human carcinoma cell lines require lower exogenous concentrations of folinic acid for growth (Scanlon et al, 1989*a*). Thus, a cell line that relies more on endogenous folate metabolism than on exogenous contributions may be less sensitive to the cytotoxic effects of cisplatin. Consequently, FBP overexpression might be

 © Cancer Research Campaign 1997

an external signal of an internal deficiency of the cell in the use of folates, and this deficiency might have relevance in the cellular response to cisplatin as increased levels of enzymes involved in dTMP cycle are needed for DNA repair.

In our experiments in which the ovarian carcinoma cell lines were cultured at high (2.3 μM) extracellular folate concentrations (Figures 1 and 2A), folate presumably enters the cells by passive diffusion, independent of the level of FBP expression (Antony et al, 1989). Because these culture conditions ensured adequate delivery of folate to all cell lines used, the differential cisplatin responsiveness cannot be attributed to different availability of endogenous folates but instead might reflect differences in the capacity to use them. FBP expression levels have been reported to be inversely regulated by the extra- and intracellular folate concentration (Kamen et al, 1986; Kane et al, 1988) but, in our hands, only one (L-SKOV3) out of five ovarian carcinoma cell lines tested showed FBP up-modulation when adapted at physiological folate concentrations (20 nM) (Miotti et al, 1995). In the same study, all the cell lines showed a marked decrease in endogenous folates compared with cells grown in standard medium (2.3 μM), and the amount of total folate in the cells was in general directly proportional to the level of membrane FBP expression, consistent with the concept that intracellular folate content is maintained primarily by FBP in low-folate cultured cells (Matsue et al, 1992). Growth in low-folate medium did not change the cisplatin sensitivity of IGROV1 and SKOV3 cells in our study. Moreover, the results obtained with proliferating cells under low-folate conditions (Figure 2B) further support the notion that cell lines with higher FBP expression are more sensitive to cisplatin treatment. In addition, the differences among the $IC_{50}$ values of sensitive and resistant cells were enhanced. In particular, SKOV3 and SW626 cells became even more cisplatin-resistant than the other lines. Together, these results are in agreement with the hypothesis that low-FBP, cisplatin-resistant cells use endogenous folates more effectively than high-FBP, cisplatin-sensitive cells.

Whereas FBP transfection of cell lines that presumably have normal folate metabolism (NIH/3T3 and CHO) or that are characterized by expression of low levels of FBP (L-SKOV3) did not result in a lower $IC_{50}$, the two cell lines of epithelial origin became more resistant than the respective mock-transfected cells (Table). These data support the notion that it is not FBP expression levels per se that underlie the cell response to cisplatin. However, the possibility exists that overexpression of FBP in plasma membrane of transfected cells reduced uptake of cisplatin, thus reducing the sensitivity of the cell lines to the drug.

Our analysis of the two cisplatin-resistant variants of IGROV1 cells confirms the inverse relationship between the degree of resistance and the number of FBP receptors (Figure 3A). This finding suggests that cisplatin treatment selects for cells that require only minimal FBP expression on the membrane for survival. This decreased FBP expression appears to be specific as other markers (Figure 3B, C and D) remained essentially unchanged or increased compared with parental cells. It has to be noted that the increased expression of HER-2/neu on resistant cells is consistent with previous observations, suggesting the cisplatin resistance of HER-2/neu-positive tumours in vivo (Berchunck et al, 1990). Thus, the in vitro selection procedure appears to result in cisplatin-resistant cells that reflect the in vivo situation.

The results of our study suggest the potential interest of FBP expression as a predictive factor in the outcome of ovarian carcinoma treatment with cisplatin, as higher levels of expression were significantly associated with more effective drug responsiveness. In addition, the data on the IGROV1-derived cisplatin-resistant cell lines suggest that cisplatin therapy can select for subpopulations of intrinsically drug-resistant tumour cells characterized by a lower FBP expression than the original tumour.

### ACKNOWLEDGEMENTS

This work was partly supported by AIRC/FIRC. We thank Mrs A Filippini for technical assistance, Mrs L Mameli for preparing the manuscript and Mr M Azzini for graphics support.

### REFERENCES

Andrews PA and Howell SB (1990) Cellular pharmacology of cisplatin: perspectives on mechanisms of acquired resistance. *Cancer Cells* **2**: 35–43

Andrews PA, Murphy MP and Howell SB (1987) Metallothionein-mediated cisplatin resistance in human ovarian carcinoma cells. *Cancer Chemother Pharmacol* **19**: 149–154

Andrews PA, Jones JA, Varki NM and Howell SB (1990) Rapid emergence of acquired cis-diamminedichloroplatinum(II) resistance in an in vivo model of human ovarian carcinoma. *Cancer Commun* **2**: 93–100

Antony AC (1992) The biological chemistry of folate receptors. *Blood* **79**: 2807–2820

Antony AC, Kane MA, Krishnan SR, Kincade RS and Verma RS (1989) Folate (pteroylglutamate) uptake in human red blood cells, erythroid precursors and KB cells at high extracellular folate concentrations. *Biochem J* **260**: 401–411

Berchunck A, Kamel A, Whitaker R, Kerns B, Olt G, Kinney R, Soper JT, Dodge R, Clarke-Pearson DL, Marks P, McKenzie S, Yin S and Bast RC JR (1990) Overexpression of HER-2/neu is associated with poor survival in advanced epithelial ovarian cancer. *Cancer Res* **50**: 4087–4091

Bottero F, Tomassetti A, Canevari S, Miotti S, Ménard S and Colnaghi MI (1993) Gene transfection and expression of the ovarian carcinoma marker folate binding protein on NIH/3T3 cells increases cell growth in vitro and in vivo. *Cancer Res* **53**: 5791–5796

Bottero F, Tomassetti A, Miotti S, Colnaghi MI and Canevari S (1995) Relevance of SER234 on functionality on the high affinity folate receptor. *FEBS Lett* **95**: 31.3

Branda RF and Blickensderfer DB (1993) Folate deficiency increases genetic damage caused by alkylating agents and gamma-irradiation in Chinese hamster ovary cells. *Cancer Res* **53**: 5401–5408

Campbell IG, Jones TA, Foulkes WD and Trowsdale J (1991) Folate-binding protein is a marker for ovarian cancer. *Cancer Res* **51**: 5329–5338

Coney LR, Tomassetti A, Carayannopoulos L, Frasca V, Kamen BA, Colnaghi MI and Zurawski VR (1991) Cloning of a tumor-associated antigen: MOv18 and MOv19 antibodies recognize a folate-binding protein. *Cancer Res* **51**: 6125–6132

Dall'Agnese L, Viel A, Capozzi E and Boiocchi M (1995) The methylenetetrahydrofolate reductase gene mapping on 1p36.3 is frequently deleted in ovarian carcinoma. *Tumori* **81**: 366

Eastman A and Schulte N (1988) Enhanced DNA repair as a mechanism of resistance to cis-diamminedichloroplatinum(II). *Biochemistry* **27**: 4730–4734

Foulkes WD, Campbell IG, Stamp GWH and Trowsdale J (1993) Loss of heterozygosity and amplification on chromosome 11q in human ovarian cancer. *Br J Cancer* **67**: 268–273

Gross RB and Scanlon KJ (1986) Amino acid membrane transport properties of L1210 cells resistant to cisplatin. *Chemioterapia* **V**: 37

Henderson GB (1990) Folate-binding proteins. *Annu Rev Nutr* **10**: 319–335

Jekunen AP, Hom DK, Alcaraz JE, Eastman A and Howell SB (1994) Cellular pharmacology of dichloro(ethylenediamine)platinum(II) in cisplatin-sensitive and resistant human ovarian carcinoma cells. *Cancer Res* **54**: 2680–2687

Kamen BA and Capdevila A (1986) Receptor-mediated folate accumulation is regulated by the cellular folate content. *Proc Natl Acad Sci USA* **83**: 5983–5987

Kane MA, Elwood PC, Portillo RM, Antony AC, Najfeld V and Finley A (1988) Influence on immunoreactive folate-binding proteins of extracellular folate concentration in cultured human cells. *J Clin Invest* **81**: 1398–1406

Loh SY, Mistry P, Kelland LR, Abel G and Harrap KR (1992) Reduced drug accumulation as a major mechanism of acquired resistance to cisplatin in a human ovarian carcinoma cell line: circumvention studies using novel platinum (II) and (IV) ammine/amine complexes. *Br J Cancer* **66**: 1109–1115

Lu Y, Han J and Scanlon KJ (1988) Biochemical and molecular properties of cisplatin-resistant A2780 cell grown in folinic acid. *J Biol Chem* **263**: 4891–4894

Masuda H, Ozols RF, Lai G-M, Fojo A, Rothenberg M and Hamilton TC (1988) Increased DNA repair as a mechanism of acquired resistance to cis-diamminedichloroplatinum(II) in human ovarian cancer cell lines. *Cancer Res* **48**: 5713–5716

Matsue H, Rothberg KG, Takashima A, Kamen BA, Anderson RGW and Lacey SW (1992) Folate receptor allows cells to grow in low concentrations of 5-methyltetrahydrofolate. *Proc Natl Acad Sci USA* **89**: 6006–6009

Miotti S, Canevari S, Ménard S, Mezzanzanica D, Porro G, Pupa SM, Regazzoni M, Tagliabue E and Colnaghi MI (1987) Characterization of human ovarian carcinoma-associated antigens defined by novel monoclonal antibodies with tumor-restricted specificity. *Int J Cancer* **39**: 297–303

Miotti S, Alberti S, Facheris P, Mantovani L, Fornaro M, Stella M, Ménard S, Canevari S and Colnaghi MI (1992) Membrane association and shedding of the GPI-anchored Ca-MOv18 antigen in human ovary carcinoma cells. *Int J Cancer* **51**: 499–505

Miotti S, Facheris P, Tomassetti A, Bottero F, Bottini C, Ottone F, Colnaghi MI, Bunni MA, Priest DG and Canevari S (1995) Growth of ovary carcinoma cell lines at physiological folate concentration: effect on folate binding protein expression in vitro and in vivo. *Int J Cancer* **63**: 395–401

Misawa T, Kikkawa F, Maeda O, Obata NH, Higashide K, Suganuma N and Tomoda Y (1995) Establishment and characterization of acquired resistance to platinum anticancer drugs in human ovarian carcinoma cells. *Jpn J Cancer Res* **86**: 88–94

Mistry P, Kelland LR, Abel G, Sidhar S and Harrap KR (1991) The relationships between glutathione-S-transferase and cytotoxicity of platinum drugs and melphalan in eight human ovarian carcinoma cell lines. *Br J Cancer* **64**: 215–220

Morrow CS and Cowan KH (1990) Glutathione-S-transferases and drug resistance. *Cancer Cells* **2**: 15–22

Nakagawa M, Nomura Y, Kohno K, Ono M, Mizoguchi H, Ogata J and Kuwano M (1993) Reduction of drug accumulation in cisplatin-resistant variants of human prostatic cancer PC-3 cell line. *J Urol* **150**: 1970–1973

Newman EM, Lu Y, Kashani-Sabet M, Kesavan V and Scanlon KJ (1988) Mechanisms of cross-resistance to methotrexate and 5-fluorouracil in an A2780 human ovarian carcinoma cell subline resistant to cisplatin. *Biochem Pharmacol* **37**: 443–447

Ozols RF (1992) Ovarian cancer. Part II: Treatment. *Curr Probl Cancer* **16**: 67–126

Ozols RF and Young RC (1991) Chemotherapy of ovarian cancer. *Semin Oncol* **18**: 222–232

Rosenberg B (1985) Fundamental studies with cisplatin. *Cancer* **55**: 2303–2316

Scanlon KJ and Kashani-Sabet M (1988) Elevated expression of thymidylate synthase cycle genes in cisplatin-resistant human ovarian carcinoma A2780 cells. *Proc Natl Acad Sci USA* **85**: 650–653

Scanlon KJ, Newman EM, Lu Y and Priest DG (1986) Biochemical basis for cisplatin and 5-fluorouracil synergism in human ovarian carcinoma cells. *Proc Natl Acad Sci USA* **83**: 8923–8925

Scanlon KJ, Kashani-Sabet M, Miyachi H, Sowers LC and Rossi J (1989*a*) Molecular basis of cisplatin resistance in human carcinomas: model systems and patients. *Anticancer Res* **9**: 1301–1312

Scanlon KJ, Kashani-Sabet M and Miyachi H (1989*b*) Differential gene expression in human cancer cells resistant to cisplatin. *Cancer Invest* **7**: 581–587

Shionoya S, Lu Y and Scanlon KJ (1986) Properties of amino acid transport systems in K562 cells sensitive and resistant to cis-diamminedichloroplatinum(II). *Cancer Res* **46**: 3445–3448

Tagliabue E, Centis F, Campiglio M, Mastroianni A, Martignone S, Pellegrini R, Casalini P, Lanzi C, Ménard S and Colnaghi MI (1991) Selection of monoclonal antibodies which induce internalization and phosphorylation of p185$^{HER2}$ and growth inhibition of cells with HER2/*neu* gene amplification. *Int J Cancer* **47**: 933–937

Tedeschi M, Bohm S, Di Re F, Oriana S, Spatti GB, Tognella S and Zunino F (1990) Glutathione and detoxification. *Cancer Treat Rev* **17**: 203–208

Tosi E, Valota O, Negri DRM, Adobati E, Mazzoni A, Meazza R, Ferrini S, Colnaghi MI and Canevari S (1995) Anti-tumor efficacy of an anti-epidermal growth factor receptor monoclonal antibody and its F(ab′)2 fragment against high- and low-EGFR-expressing carcinomas in nude mice. *Int J Cancer* **62**: 643–650

© Cancer Research Campaign 1997